# Objective response to immune checkpoint inhibitor therapy in *NRAS*-mutant melanoma: A systematic review and meta-analysis

**DOI:** 10.3389/fmed.2023.1090737

**Published:** 2023-02-16

**Authors:** Zachary J. Jaeger, Neel S. Raval, Natalia K. A. Maverakis, David Y. Chen, George Ansstas, Angela Hardi, Lynn A. Cornelius

**Affiliations:** ^1^Office of Medical Student Education, Washington University School of Medicine, St. Louis, MO, United States; ^2^San Juan Bautista School of Medicine, Caguas, PR, United States; ^3^Alvin J. Siteman Comprehensive Cancer Center, Washington University School of Medicine, St. Louis, MO, United States; ^4^Division of Dermatology, Department of Medicine, Washington University School of Medicine, St. Louis, MO, United States

**Keywords:** metastatic melanoma, cutaneous melanoma, immunotherapy, checkpoint inhibitor, NRAS, objective response rate, disease control rate, NRAS-mutant melanoma

## Abstract

**Introduction:**

*NRAS* mutations are common in melanoma and confer a worse prognosis. Although most patients with metastatic melanoma receive immune checkpoint inhibitors (ICIs), the impact of *NRAS* mutational status on their efficacy remains under debate.

**Methods:**

We performed a comprehensive literature search across several large databases. Inclusion criteria were trials, cohorts, and large case series that analyzed the primary outcome of objective response rate by *NRAS* mutational status in patients with melanoma treated with any line of ICI. At least two reviewers independently screened studies using Covidence software, extracted data, and assessed risk of bias. Standard meta-analysis was performed in R with sensitivity analysis and tests for bias.

**Results:**

Data on 1770 patients from ten articles were pooled for meta-analysis, and the objective response rate to ICIs was calculated to compare *NRAS*-mutant and *NRAS*-wildtype melanoma. The objective response rate was 1.28 (95% confidence interval: 1.01–1.64). Sensitivity analysis identified the study by Dupuis et al. with influential impact on the pooled effect size and heterogeneity, favoring *NRAS*-mutant melanoma.

**Discussion:**

In this meta-analysis evaluating the impact of *NRAS* mutational status on objective response to ICIs in metastatic melanoma, *NRAS*-mutant cutaneous melanoma demonstrated an increased likelihood of partial or complete tumor response, relative to *NRAS*-wildtype cutaneous melanoma. Genomic screening for *NRAS* mutations in patients with metastatic melanoma may improve predictive ability when initiating ICIs.

## 1. Introduction

Melanoma is the sixth-leading cause of cancer diagnosis in the United States and is responsible for the most deaths from skin cancer (1). The Surveillance, Epidemiology, and End Results database indicates that incidence of cutaneous melanoma has increased over the past two decades (2). Whereas FDA approval of multiple immune checkpoint inhibitors (ICIs) in the past decade has revolutionized therapy, five-year survival still hovers around 30% for metastatic melanoma (2–6). While new strategies are being investigated, we are interested in understanding whether and why molecular subtypes of melanoma may respond differently to ICIs (7).

The contemporaneous emergence of next-generation sequencing has improved our abilities to characterize individual patients’ melanomas and to research potential genetic targets. *NRAS* mutations are present in approximately 20% of all melanomas and portend a worse prognosis than does *NRAS*-wildtype status (8, 9). Although the lack of effective targeted therapy makes *NRAS*-mutant melanoma a more controversial subtype than *BRAF*-mutant melanoma, studies suggest that *NRAS* mutational status is an independent prognostic factor for metastatic melanoma and may be associated with immunotherapy response (9–15). Immunotherapy is considered the standard of care for most patients with locally advanced or metastatic melanoma without a contraindication, but their comparative efficacy in *NRAS*-mutant versus *NRAS*-wildtype melanoma remains unclear (9, 10). Some studies have suggested that in advanced melanoma treated with ICIs, *NRAS*-mutation-positive status is associated with higher rates of tumor objective response and/or prolonged survival (16, 17), while others do not support this association (18, 19). To our knowledge, a comprehensive synthesis of the available evidence of the outcomes of ICIs for melanoma based on *NRAS* mutational status has not been undertaken.

Therefore, we conducted a systematic review and meta-analysis to critically appraise and synthesize the published data comparing objective response rate (ORR) to ICIs by *NRAS* mutational status in patients with melanoma.

## 2. Manuscript

### 2.1. Methods

This systematic review was registered in the PROSPERO database (ID: CRD42021273588) and reported according to PRISMA and MOOSE guidelines. The Washington University Institutional Review Board determined this project to be non-human subjects research.

#### 2.1.1. Search strategy

A clinical librarian (AH) executed a comprehensive search in PubMed, Embase, Scopus, Web of Science, Cochrane Library (including CENTRAL), and Clinicaltrials.gov databases from database origin to 14 June 2022. The full search strategy is available in the Supplementary Materials. Results were limited to English and human studies.

Two reviewers independently screened titles and abstracts for eligibility (ZJJ, NSR) and, subsequently, relevant full texts (ZJJ, NSR) using the Covidence systematic review management program (20). One reviewer performed forward and reverse citation searches (ZJJ), i.e., screenings of articles citing and cited by included studies, respectively. Two or three reviewers independently extracted data (ZJJ, NSR, NKAM). Disagreements were resolved by consensus.

#### 2.1.2. Eligibility criteria

We included randomized, controlled trials and cohort studies that analyzed ORR by *NRAS* mutational status in patients with melanoma treated with any line of ICI. We excluded unpublished work, gray literature, duplicate studies, animal studies, *in vitro* studies, case reports, expert opinions, and reviews.

The primary outcome was ORR for *NRAS*-mutant and *NRAS*-wildtype melanoma, defined as the percentage of patients with complete (CR) or partial response (PR) by Response Evaluation Criteria in Solid Tumors v1.1 or immune-related response criteria at the time of last follow-up (21, 22). We defined the comparison group as either confirmed to be triple-negative for *NRAS*, *BRAF*, and *NF1* mutations or at least confirmed *NRAS*-wildtype; if comparison groups included melanoma with other mutations, previous or current targeted therapy merited exclusion. If the genotype data were not explicitly stated, we contacted authors for clarification. Patients with stable (SD) or progressive disease (PD) were considered unresponsive to treatment. A secondary outcome was the disease control rate (DCR), denoted by the percentage of patients with CR, PR, or SD. Other extracted data included patient sex and age, melanoma subtype, stage at ICI initiation, ICI class and line, and tumor mutational burden (TMB).

#### 2.1.3. Quality assessment

Three reviewers (ZJJ, NSR, and NKAM) independently assessed the overall quality of evidence of each study *via* the Oxford Centre for Evidence-Based Medicine (OCEBM) Levels of Evidence and modified Newcastle-Ottawa scale (mNOS) (23, 24). We designated OCEBM study quality per the “Therapy/Prevention, Aetiology/Harm” category from 1a (best) to 5 (poorest), with mNOS scored from 0 (poorest) to 9 (best). To test for potential publication bias, we visually inspected a contour-enhanced funnel plot, graphically evaluated using the trim-and-fill method (25), and statistically evaluated with the Egger regression test (26).

#### 2.1.4. Data synthesis and statistical analysis

We used R version 4.1.2 (packages “metafor” and “metaviz”) for all data analysis. We performed a meta-analysis of the pooled relative risk ratios of ORR of cutaneous melanoma using a random-effects model. The Yates correction was employed for cells with zero to estimate the 95% confidence intervals (CI). The results were plotted on a forest plot with 95% CI of the relative risk ratios of ORR. We evaluated heterogeneity between studies with the *I^2^* statistic (greater than 50% suggesting moderate heterogeneity) and Q statistic (greater than the degrees of freedom suggesting significant heterogeneity). We repeated these methods for DCR. A sensitivity analysis was performed to identify influential studies in the ORR meta-analysis.

### 2.2. Results

#### 2.2.1. Literature search

**Figure 1:**
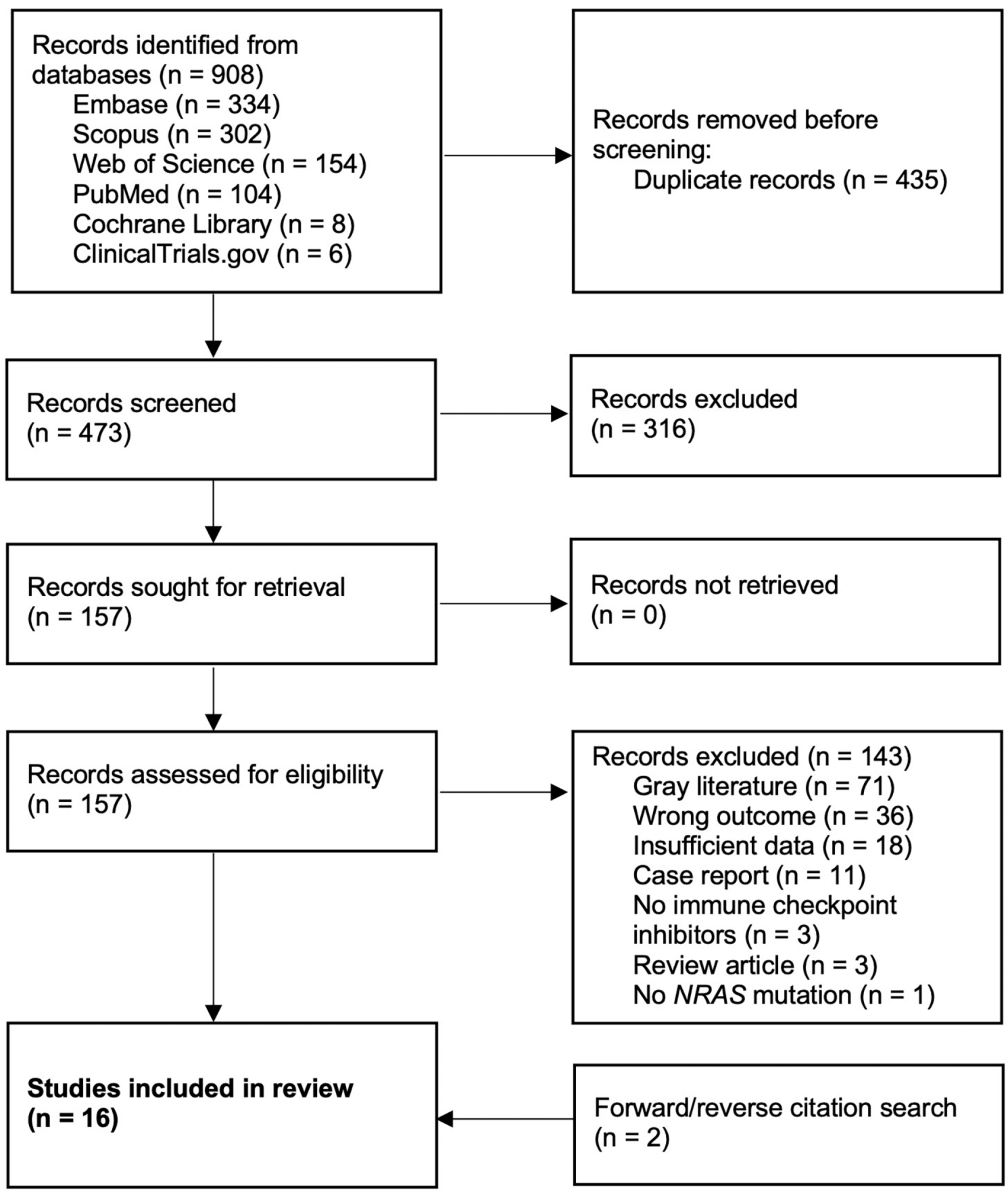
PRISMA flow diagram.

After removal of duplicates, 473 records were screened, 157 full-text articles were assessed, and 16 studies met inclusion criteria for the systematic review, with 10 of those being amenable to meta-analysis (Figure 1) (16, 18, 19, 27–39). Data on 1770 patients from two prospective studies and eight retrospective cohort studies were pooled for analysis.

#### 2.2.2. Study characteristics

Most included studies were retrospective cohorts and took place in the 2010s in North America, Europe, and Asia (Table 1). Three of four prospective trials were carried out in Asia, with the other trial by Ascierto et al. occurring in Italy (27, 33, 35, 38). Participants were generally middle-aged adults with an approximate pooled median of 55–65 years, with a slight male predominance (54%). Cutaneous melanoma was the predominant subtype in 11 studies, with varying prevalence of non-cutaneous subtypes (mucosal, acral, uveal, and unknown primary) comprising the remainder. Most of the studies of patients with non-cutaneous melanoma occurred in Asia; these studies were included for the systematic review but excluded from meta-analysis (33, 35, 36, 38). All studies involved patients with metastatic melanoma, many with predominantly Stage IV (78% among those reporting stage). ICI regimens varied, ranging from anti-CTLA-4 (8/16, 50%) or anti-PD-1/PD-L1 monoclonal antibody (14/16, 88%) monotherapy to combinations thereof (5/16, 31%), some combined with chemotherapy (3/16, 19%). Only five studies included patients on combination ICI therapy, ranging from 1 to 40% of the study population. Data were unavailable for individual participants by mutational status, precluding subgroup analysis by ICI line and class.

**Table 1:** Summary data and quality assessment.

Author	Year	Country	Study type	Female, *n* (%)	Age (in years)	Predominant melanoma type	Class of ICI^a^	OCEBM/mNOS Quality^b^
Ascierto et al.	2014	Italy	Prospective cohort	395 (46)	Median 61, Min–max^c^ 16–88	Cutaneous	Anti-CTLA-4 monotherapy	2b/7
Lipson et al.	2014	United States	Case series	4 (50)	Median 58, Min–max 43–79	Cutaneous	Anti-CTLA-4 or anti-PD-L1 monotherapy	4/4
Johnson et al.	2015	United States	Retrospective cohort	50 (28)	61% 60+	Cutaneous	Anti-PD-1, anti-PD-L1, anti-CTLA-4, or IL-2 monotherapy	2b/8
Johnson et al.	2016	United States	Retrospective cohort	27 (42)	Initial: Median 55, Min-max 33–80; Validation: Median 62, Min-max 32–85	Cutaneous	Anti-PD-1 or anti-PD-L1 monotherapy	2b/8
Jamal et al.	2017	Canada	Prospective cohort	8 (27)	Median 55, Min–max 26–74	Cutaneous	Anti-CTLA-4 monotherapy	2b/7
Afzal et al.	2018	United States	Retrospective cohort	21 (38)	Mean 63, SD^d^ 15	Cutaneous	Anti-PD-1 monotherapy or anti-CTLA-4/anti-PD-1 combination therapy	2b/9
Dupuis et al.	2018	France	Retrospective cohort	29 (41)	61% 60+	Cutaneous	Anti-PD-1 monotherapy	2b/8
Kirchberger et al.	2018	Germany	Retrospective cohort	154 (42)	Median 64, Min–max 20–87	Cutaneous	Anti-CTLA-4 or anti-PD-1 monotherapy or combination therapy	2b/8
Sheng et al.	2019	China	Phase 1B clinical trial	20 (61)	Median 54, Min–max 27–70	Mucosal	Anti-PD-1 monotherapy	2b/7
Loo et al.	2020	United States	Retrospective cohort	22 (33)	Median 59, Min–max 27–93	Cutaneous	Anti-CTLA-4 or anti-PD-1 monotherapy or combination therapy	2b/7
Tang et al.	2020	China	Phase 2 clinical trial	70 (55)	Mean 52, SD 11	Acral, mucosal	Anti-PD-1 monotherapy	2b/7
Byeon et al.	2021	Korea	Retrospective cohort	90 (51)	Median 61, Min–max 23–84	Acral, cutaneous, mucosal	Anti-PD-1 monotherapy	2b/8
Guida et al.	2021	Italy	Retrospective cohort	127 (38)	*NRAS*+: Median 63, IQ^e^ min–max 53–74; *NRAS*-: Median 65, IQ min–max 54–73	Cutaneous	Anti-PD-1 or anti-CLTA-4 monotherapy or combination therapy	2b/9
Li et al.	2021	China	Retrospective cohort	32 (50)	Median 54, Min–max 25–72	Cutaneous	Anti-PD-1 monotherapy	2b/8
Zhou et al.	2021	China	Prospective trials	119 (58)	15% 65+	Non-cutaneous, cutaneous	Anti-PD-1 monotherapy	2b/9
Zhang et al.	2022	International	Retrospective cohort	199 (36)	51% 61+	Cutaneous	Anti-CTLA-4, anti-PD-1, or anti-PD-L1 monotherapy or combination therapy	2b/8

^a^ICI: immune checkpoint inhibitor. ^b^OCEBM Quality Assessment: Oxford Centre for Evidence-based Medicine Levels of Evidencem; NOS: modified Newcastle-Ottawa scale. ^c^Min–max: minimum to maximum. ^d^SD: standard deviation. ^e^IQ: interquartile.

#### 2.2.3. Meta-analysis

**Figure 2:**
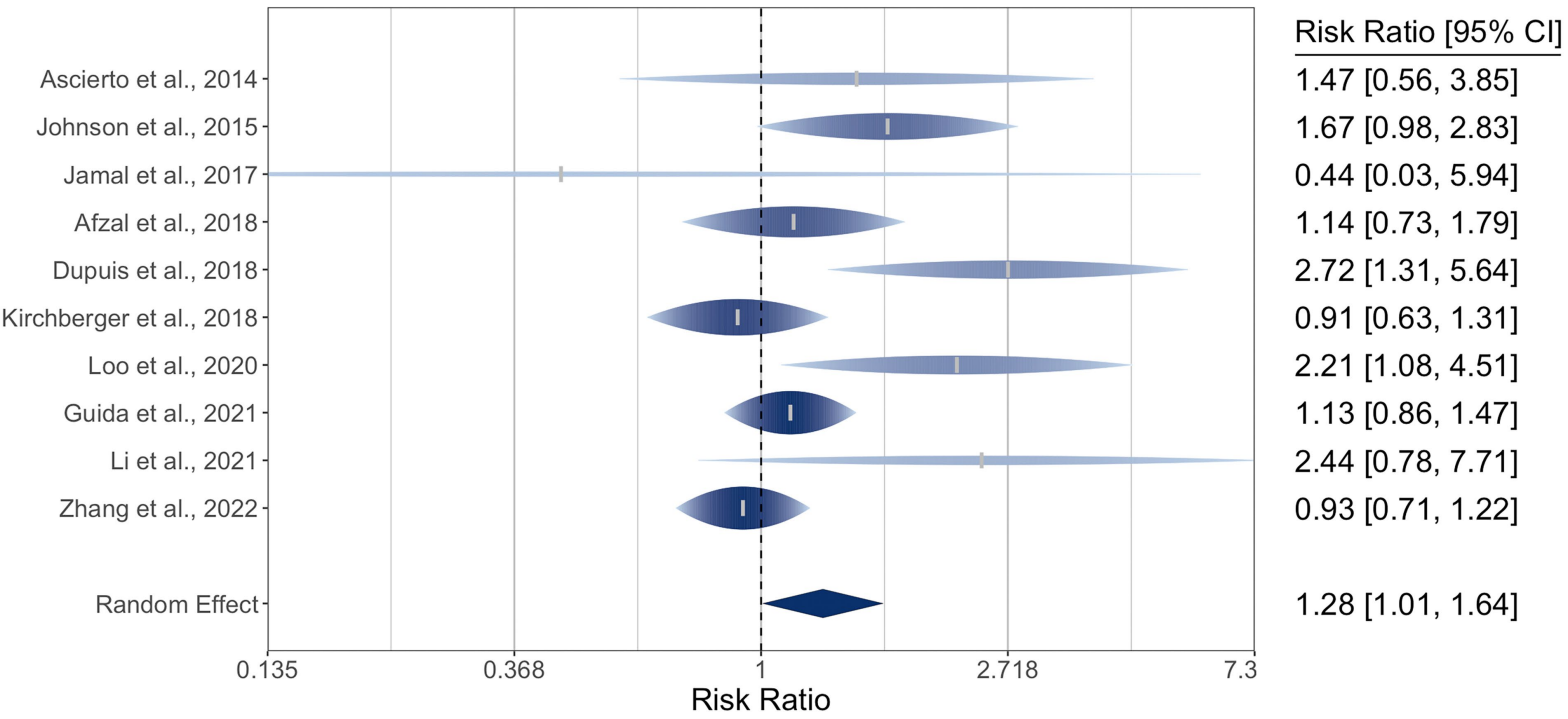
Forest plot of the random-effects meta-analysis for relative risk ratios of objective response rates (ORR) of cutaneous melanoma by *NRAS* mutational status. *NRAS*-mut: *NRAS*-mutant melanoma; *NRAS*-wt: *NRAS*-wildtype melanoma; OR: Objective response; NR: No objective response.

For the ten included studies encompassing 1770 participants, the pooled relative risk ratio (RR) of ORR by *NRAS* mutational status was 1.28 (95% CI: 1.01–1.64). The *I^2^* statistic was 53%, indicating moderate-to-high heterogeneity, and Q (*df* = 9) was 17 (*p* = 0.05). The meta-analysis of ORR is graphically represented in a rainforest plot in Figure 2.

**Figure 3:**
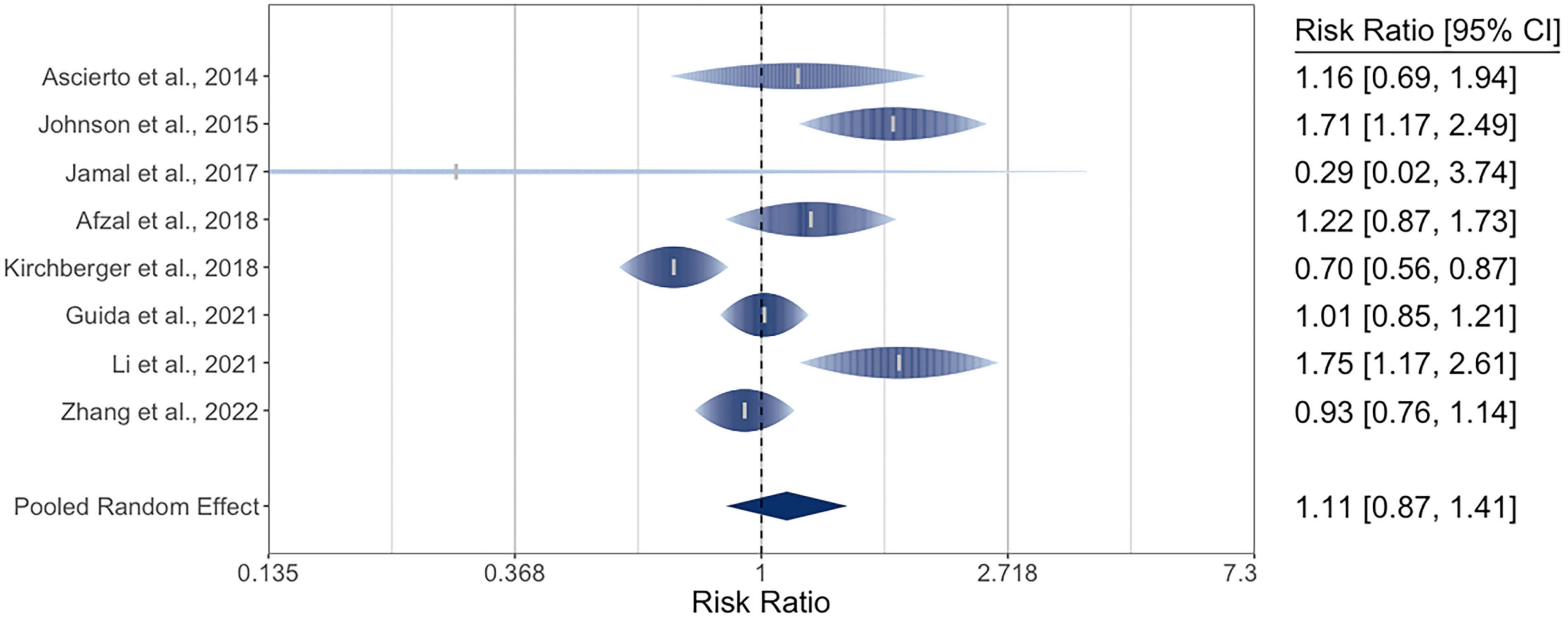
Forest plot of the random-effects meta-analysis for relative risk ratios of disease control rates (DCR) of cutaneous melanoma by *NRAS* mutational status. *NRAS*-mut: *NRAS*-mutant melanoma; *NRAS*-wt: *NRAS*-wildtype melanoma; DC: Disease control; PD: Progressive disease.

There were eight studies reporting DCR for 1,650 patients with metastatic cutaneous melanoma. The pooled RR by *NRAS* mutational status was 1.11 (95% CI: 0.87–1.41). There was moderate-to-high heterogeneity, with *I^2^* at 79% (95% CI: 44–97) and Q (*df* = 7) at 28 (*p* = 0.0002). The three studies in the ORR meta-analysis that did not provide DCR data reported RRs of 1.70, 2.21, and 2.72, favoring *NRAS*-mutant melanoma (29, 32, 34). The meta-analysis of DCR is graphically represented in a rainforest plot in Figure 3.

#### 2.2.4. Sensitivity analysis

Leave-one-out sensitivity analysis of ORR produced overall effect sizes ranging from 1.16 to 1.38 (lower bound of 95% CI: 0.96–1.05; upper bound: 1.40–1.83). The *I^2^* statistic ranged from 27 to 61%, and Q ranged from 11.3 to 16.9. Omission of the study by Zhang et al. maximized the lower bound at 1.05, greater than the null RR of 1 (39). Individual omission of the study by Dupuis et al. minimized *I^2^* to 27% and the 95% CI bounds to 0.96 and 1.40 (26); it also minimized the pooled estimate to 1.16(), while the remainder ranged from 1.20() to 1.38 (32, 34, 39).

#### 2.2.5. Quality assessment and risk of publication bias

Oxford Centre for Evidence-Based Medicine quality ratings (Table 1) ranged from 2b to 4, with all earning a grade of 2b except the case series (28). mNOS ranged from 7 to 9 indicating generally low risk of bias, except for the case series (4). Inspection of the contour-enhanced funnel plot of the ORR meta-analysis revealed some asymmetry, with larger studies tending to report positive effect sizes and one small study reporting a non-significant negative result. The trim-and-fill method imputed two moderate-to-large studies, one with a non-significant negative effect size, the other on the borderline of significance, and the Egger regression test was statisticallysignificant for asymmetry (*p* = 0.02). The Baujat plot identified the study by Dupuis et al. (32, 40) and as particularly influential on the overall results. Graphical representations of statistical tests for bias are included in the Supplementary material.

#### 2.2.6. Tumor mutational burden

Six studies reported outcome data on TMB (29, 33, 35–37, 39). Four of these reported increased ORR in higher-TMB groups ranging from 30 to 85%, with only Johnson et al.’s (29, 33, 35–37) paper achieving statistical significance (85%, *p* < 0.001). Byeon et al. (36) reported better one-year progression-free survival for the higher-TMB group on ICIs at approximately 45 vs. 30%, though it was not quite statistically significant (*p* = 0.051). Zhang et al. (39) found higher TMB associated with prolonged overall survival (*p* < 0.001).

### 2.3. Discussion

The seminal paper by Johnson et al. (16) in 2015 supported positive *NRAS* mutational status as a predictor of good response to ICIs. In 2018, Dupuis et al. (32) also found an increased ORR for *NRAS*-mutant melanoma. That same year, however, Kirchberger et al. (18) demonstrated equivocal predictive value of *NRAS* mutational status. In 2021, Guida et al. (19) explored this hypothesis further and discovered a similar questionable effect with non-significant confidence intervals. In contribution to the debate surrounding the effect of the *NRAS* mutational status of melanoma on ICI response, this meta-analysis demonstrated a benefit for *NRAS*-mutant cutaneous melanoma (RR 1.28), i.e., a higher ICI response rate was seen for *NRAS*-mutant melanoma relative to wildtype. Implications of this conclusion could include, for instance, increased pertinence of *NRAS* mutational status ordering for patients with newly diagnosed metastatic cutaneous melanoma, or increased likelihood of initiating ICIs in metastatic cutaneous melanoma found to harbor an *NRAS* mutation. Further research into the potential immunological mechanism should continue to be explored. Interestingly, the results for DCR were lower and centered around the null; this could be partially explained by the exclusion of three studies with positive RR for ORR that did not report data for DCR.

Quality ratings of included studies were consistent with observational data. The funnel plot and trim-and-fill method indicated moderate concern for significant publication bias, and the Egger regression test supported the presence of asymmetry. Sources of bias such as heterogeneity between subgroups, selective outcome reporting, or chance, could be contributing to asymmetry of the funnel plot (41). In further exploration of heterogeneity, the Baujat plot indicated that the study by Dupuis et al. exerted disproportionate influence on the meta-analysis, and the sensitivity analysis was consistent with its impact on both the pooled effect size and heterogeneity measures (32). Taken together, the overall certainty of the evidence for ORR was rated as moderate (42).

The included studies largely assessed cutaneous melanoma patients based in Europe and the United States and excluded non-cutaneous melanoma studies due to their relative paucity and to avoid unlike comparisons. The few non-cutaneous-predominant studies by Sheng et al., Tang et al., Byeon et al., and Zhou et al. (33, 35, 36, 39) generally reported low ORR for *NRAS*-mutant melanoma. It is well known that non-cutaneous melanoma subtypes carry unique mutation patterns distinct from ultraviolet radiation-related signatures and often respond poorly to current therapies (43–45). Further investigation of this diverse population is warranted to better guide therapy and improve outcomes (46, 47).

The collected literature offers several potential explanations for the observed effects of ICIs on *NRAS*-mutant melanoma. Johnson et al. conducted a separate analysis which found higher PD-L1 expression in *NRAS*-mutant melanoma, though this failed to reach significance and other larger cohorts have not found PD-L1 expression to necessarily be consequential to ICI response (16, 48). In contrast, Byeon et al. (36) speculated *NRAS*-mutant melanoma could be associated with ICI resistance, perhaps due to alterations in cell surface proteins necessary for T-cell response similar to *TP53*-mutant tumors. Still others have hypothesized a synergistic effect of MAP kinase and ICI due to greater melanoma antigen production, though these preclinical findings need further real-world confirmation (49). Interestingly, in a subset of *NRAS*-mutant melanoma patients receiving immunotherapy, a frequently co-occurring PBAF complex mutation was associated with greater progression-free and overall survival (50). Most frequently, however, authors posit that TMB may be central to the ICI-*NRAS* interaction.

Quantifying TMB has become more critical in understanding cancer therapeutics and may help predict ICI response. In theory, higher TMB produces a greater degree of passenger mutations, increasing tumor neoantigenicity, T-lymphocyte infiltration, and antitumor immune response (7, 48, 51). A large-scale retrospective analysis of several primary tumor types found that TMB status may explain 55% of ORR variation to ICIs (52). Similarly, CheckMate 067 trial data revealed ICI responders were more likely to have higher TMB and higher inflammatory signatures than non-responders (53). Six studies included in this meta-analysis reported TMB data associating higher TMB with higher ORR, though an accurate pooled effect cannot be calculated here (29, 33, 35–37, 39). A meta-analysis that focuses on the role of TMB on melanoma outcomes in patients initiating ICIs is needed.

*NRAS*-mutant melanoma has proven challenging to treat, especially for those unresponsive to immunotherapy. The NRAS protein itself is difficult to target directly, and upstream and downstream effectors have demonstrated equivocal efficacy, with a few showing promise (9). Although testing is available from commercial entities, it may not be routinely performed due to a lack of effective target therapy. MEK inhibitors, alone or in combination with pan-RAF, CDK4/6, or focal adhesion kinase inhibitors, are under investigation in clinical trials for metastatic *NRAS*-mutant melanoma (14). Others are studying the predictive value of biomarkers and genomic profiles that might shed light on subpopulations more or less responsive to certain immunotherapy regimens (54, 55). One study identified *NF1* mutational status as a predictor of poor response (56), while another found good tumor response in *NF1*-mutant melanoma (57); clearly, there is a need for further investigation. Indeed, the field of melanoma continues to head toward a “multiomics”-driven approach to mutational and molecular-level stratification that could reveal predictors of therapeutic response or novel targets and treatments (58–60).

#### 2.3.1. Limitations

The review presented several challenges. Despite a sensitive literature search, the dearth of eligible articles, combined with the novelty of ICIs, resulted in a relatively small number of included studies. Some heterogeneity among variables and outcomes of interest was present, leading to possible reporting bias. This was partially mitigated by some authors responding to share supplementary data, but with more administrative resources, a meta-analysis with individual participant data and meta-regression would better characterize subgroups and adjust for potentially confounding variables.

#### 2.3.2. Conclusion

Melanoma is a leading cause of cancer diagnosis in the United States and a diagnosis that dermatologists frequently encounter in both inpatient and outpatient settings. At high stages, prognosis is poor, and even more so when *NRAS* mutational status is positive. In this meta-analysis comparing objective response to ICI therapy between *NRAS*-mutant and *NRAS*-wildtype metastatic melanoma, there was an increased likelihood of response for *NRAS*-mutant cutaneous melanoma. Genomic screening for patients diagnosed with metastatic melanoma may improve predictive ability for those harboring *NRAS* mutations. In the era of precision medicine with checkpoint inhibition and combination therapies, more prospective research on all types of melanoma is warranted, especially randomized, controlled trials on *NRAS* mutational status reporting complete demographics, molecular data, tumor response, and survival outcomes.

## Data availability statement

Publicly available datasets were analyzed in this study. This data can be found at: https://pubmed.ncbi.nlm.nih.gov/24885479/; https://pubmed.ncbi.nlm.nih.gov/33399091/; https://www.ncbi.nlm.nih.gov/pmc/articles/PMC6048096/; https://pubmed.ncbi.nlm.nih.gov/33530579/; https://pubmed.ncbi.nlm.nih.gov/29157311/; https://www.ncbi.nlm.nih.gov/pmc/articles/PMC4351797/; https://pubmed.ncbi.nlm.nih.gov/27671167/; https://pubmed.ncbi.nlm.nih.gov/29843107/; https://pubmed.ncbi.nlm.nih.gov/34181096/; https://pubmed.ncbi.nlm.nih.gov/25516806/; https://pubmed.ncbi.nlm.nih.gov/32564504/; https://pubmed.ncbi.nlm.nih.gov/31403867/; https://www.ncbi.nlm.nih.gov/pmc/articles/PMC8787219/; https://pubmed.ncbi.nlm.nih.gov/29966520/; https://pubmed.ncbi.nlm.nih.gov/34290710/; https://pubmed.ncbi.nlm.nih.gov/32321714/.

## Author contributions

ZJ, NR, DC, GA, and LC: study conception and design. ZJ, NR, NM, AH, and LC: data collection. ZJ, NR, NM, DC, GA, and LC: analysis and interpretation of results and draft manuscript preparation. All authors meet ICMJE criteria for authorship, and all those who meet ICMJE criteria are listed as authors. All authors contributed to the article and approved the submitted version.

## Funding

Discretionary research funds received from the Division of Dermatology, John T. Milliken Department of Medicine, Washington University School of Medicine in St. Louis.

## Conflict of interest

The authors declare that the research was conducted in the absence of any commercial or financial relationships that could be construed as a potential conflict of interest.

## Publisher’s note

All claims expressed in this article are solely those of the authors and do not necessarily represent those of their affiliated organizations, or those of the publisher, the editors and the reviewers. Any product that may be evaluated in this article, or claim that may be made by its manufacturer, is not guaranteed or endorsed by the publisher.

## Abbreviations

ICI: immune checkpoint inhibitor
ORR: objective response rate
CR: complete response
PR: partial response
SD: stable disease
PD: progressive disease
DCR: disease control rate
TMB: tumor mutational burden
CI: confidence interval
RR: relative risk
OCEBM: Oxford Centre for Evidence-based Medicine
mNOS: modified Newcastle-Ottawa scale
ALM: acral lentiginous melanoma
