# Supplementary material for: Objective response to immune checkpoint inhibitor therapy in NRAS-mutant melanoma: A systematic review and meta-analysis
Source: Front Med (Lausanne). 2023 Feb 16;10:1090737. doi: 10.3389/fmed.2023.1090737 (PMC9979544; doi:10.3389/fmed.2023.1090737)
Supplement: Supplementary file 1 [file Data_Sheet_1.pdf]

Supplementary Material

1 Supplementary Data: Systematic Review Search Methodology

Search Strategies created by Angela Hardi, MLIS; August 2021; Searches updated June 14, 2022

Methods:

The published literature was searched using strategies designed by a medical librarian for the concepts of melanomas, NRAS mutations, and immune-checkpoint inhibitors. These strategies were created using a combination of controlled vocabulary terms and keywords, and were executed in PubMed 1946-, Embase.com 1947-, Scopus 1823-, Web of Science 1900-, Cochrane Library (including CENTRAL), and Clinicaltrials.gov. Results were limited to English using database-supplied filters. A filter was also used to exclude animal-only studies from PubMed and Embase.[1] The initial search was completed on August 18, 2021, with an updated search completed on June 14, 2022. Full search strategies are provided.

Results:

A total of 713 results were retrieved from the initial database literature search and imported into Endnote. Six citations from ClinicalTrials.gov were retrieved and added to an Excel file library. Using the Bramer technique[2], 301 duplicates were identified and removed in Endnote. The citation library was imported into Covidence and two more duplicates were removed. After removing duplicates, 410 unique citations remained for analysis. The search was run again in June 2022 to check for newly published literature. A total of 195 results were retrieved with 132 duplicates, leaving 63 unique citations for analysis. Between the two searches, a total of 473 unique citations were retrieved.

References:

1. McGill Library. "Advanced tools for building the search: Search filters and hedges." Accessed February 19, 2021: https://libraryguides.mcgill.ca/knowledge-syntheses/search-tools
2. Bramer WM, Giustini D, de Jonge GB, Holland L, Bekhuis T. De-duplication of database search results for systematic reviews in EndNote. J Med Libr Assoc. 2016; 104(3):240-243. doi:10.3163/1536-5050.104.3.014

Endnote Library Stats:

Total number retrieved from database search: 713

Number of duplicates identified: 303 (including 2 in Covidence)

Number of unique citations: 410 (404 in Endnote + 6 ClinicalTrials.gov results)

Updated Search Endnote Library Stats:

Total number retrieved from database search: 195

Number of duplicates identified: 132 (98 within library, 33 between original and updated libraries, 1 in Covidence)

Number of new unique citations: 63

Number of studies from updated search included in systematic review: 2

Complete Search Strategies:

Embase.com

=267 results on 8/18/2021; Animal-only studies excluded using filter; Limited to English

Updated search (original search with date limit 2021-present) = 67 results on 6/14/2022

('melanoma'/exp OR (melanoma* OR melanocarcinoma* OR melanomalignoma* OR naevocarcinoma* OR nevocarcinoma*):ti,ab) AND ('nras gene'/exp OR (‘NRAS’ OR ‘NRAS1’ OR ‘NS6’ OR ‘N-ras’ OR ‘neuroblastoma RAS viral oncogene homolog’ OR ‘v-ras neuroblastoma RAS viral oncogene homolog’ OR ‘oncogene N ras’):ti,ab) AND ((‘immune checkpoint inhibitor'/exp OR 'ipilimumab'/exp OR 'nivolumab'/exp OR 'pembrolizumab'/exp OR 'atezolizumab'/exp OR 'avelumab'/exp OR 'durvalumab'/exp OR 'cemiplimab'/exp) OR (‘immune checkpoint inhibitor*’ OR ‘checkpoint inhibitor immunotherap*’ OR ‘immune checkpoint block*’ OR ‘immune checkpoint inhabitation*’ OR ‘PD-L1 inhibitor*’ OR ‘PD L1 inhibitor*’ OR ‘anti-PDL 1’ OR ‘programmed death ligand inhibitor*’ OR ‘programmed death-ligand 1 Inhibitor*’ OR ‘CTLA-4 inhibitor*’ OR ‘CTLA 4 inhibitor*’ OR ‘cytotoxic T-lymphocyte-associated protein 4 inhibitor*’ OR ‘cytotoxic T lymphocyte associated protein 4 inhibitor*’ OR ‘PD-1 inhibitor*’ OR ‘PD 1 inhibitor*’ OR ‘programmed cell death protein 1 inhibitor*’ OR ‘PD-1-PD-L1 blockade*’ OR ‘PD 1 PD L1 blockade*’ OR ipilimumab OR strentarga OR yervoy OR nivolumab OR opdivo OR pembrolizumab OR keytruda OR lambrolizumab OR atezolizumab OR tecentriq OR tecntriq OR avelumab OR bavencio OR durvalumab OR imfinzi OR cemiplimab OR libtayo):ti,ab) NOT ([animals]/lim NOT [humans]/lim) AND [english]/lim

PubMed

=77 results on 8/18/2021; Animal-only studies excluded using filter; Limited to English (Best Match Sort)

Updated search (original search with date limit 2021-present): 27 results on 6/14/2022

("Melanoma"[Mesh] OR melanoma*[tiab] OR melanocarcinoma*[tiab] OR melanomalignoma*[tiab] OR naevocarcinoma*[tiab] OR nevocarcinoma*[tiab]) AND ("NRAS protein, human"

[Supplementary Concept] OR “NRAS”[tiab] OR “NRAS1”[tiab] OR “NS6”[tiab] OR “N-ras”[tiab] OR “neuroblastoma RAS viral oncogene homolog”[tiab] OR “oncogene N ras”[tiab]) AND ("Immune Checkpoint Inhibitors"[Mesh] OR "Ipilimumab"[Mesh] OR "Nivolumab"[Mesh] OR "pembrolizumab" [Supplementary Concept] OR "atezolizumab" [Supplementary Concept] OR "avelumab" [Supplementary Concept] OR "durvalumab" [Supplementary Concept] OR "cemiplimab" [Supplementary Concept] OR “immune checkpoint inhibitor*”[tiab] OR “checkpoint inhibitor immunotherap*”[tiab] OR “immune checkpoint block*”[tiab] OR “PD-L1 inhibitor*”[tiab] OR “PD L1 inhibitor*”[tiab] OR “anti-PDL 1” [tiab] OR “programmed death ligand inhibitor*”[tiab] OR “programmed death-ligand 1 Inhibitor*”[tiab] OR “CTLA-4 inhibitor*”[tiab] OR “CTLA 4 inhibitor*”[tiab] OR “cytotoxic T-lymphocyte-associated protein 4 inhibitor*”[tiab] OR “cytotoxic T lymphocyte associated protein 4 inhibitor*”[tiab] OR “PD-1 inhibitor*”[tiab] OR “PD 1 inhibitor*”[tiab] OR “programmed cell death protein 1 inhibitor*”[tiab] OR “PD-1-PD-L1 blockade*”[tiab] OR “PD 1 PD L1 blockade*”[tiab] OR ipilimumab[tiab] OR yervoy[tiab] OR nivolumab[tiab] OR opdivo[tiab] OR pembrolizumab[tiab] OR keytruda[tiab] OR lambrolizumab[tiab] OR atezolizumab[tiab] OR tecentriq [tiab] OR avelumab[tiab] OR bavencio[tiab] OR durvalumab[tiab] OR imfinzi[tiab] OR cemiplimab[tiab] OR libtayo[tiab]) NOT ("Animals"[Mesh] NOT ("Animals"[Mesh] AND "Humans"[Mesh]))

Scopus

=238 results on 8/18/2021; English limit used; Book chapters and editorials excluded from results.

Updated search (original search with date limit 2021-present): 64 results on 6/14/2022

TITLE-ABS-KEY(melanoma* OR melanocarcinoma* OR melanomalignoma* OR naevocarcinoma* OR nevocarcinoma*) AND TITLE-ABS-KEY(“NRAS” OR “NRAS1” OR “NS6” OR “N-ras” OR “neuroblastoma RAS viral oncogene homolog” OR “v-ras neuroblastoma RAS viral oncogene homolog” OR “oncogene N ras”) AND TITLE-ABS-KEY(“immune checkpoint inhibitor*” OR “checkpoint inhibitor immunotherap*” OR “immune checkpoint block*” OR “immune checkpoint inhabitation*” OR “PD-L1 inhibitor*” OR “PD L1 inhibitor*” OR “anti-PDL 1” OR “programmed death ligand inhibitor*” OR “programmed death-ligand 1 Inhibitor*” OR “CTLA-4 inhibitor*” OR “CTLA 4 inhibitor*” OR “cytotoxic T-lymphocyte-associated protein 4 inhibitor*” OR “cytotoxic T lymphocyte associated protein 4 inhibitor*” OR “PD-1 inhibitor*” OR “PD 1 inhibitor*” OR “programmed cell death protein 1 inhibitor*” OR “PD-1-PD-L1 blockade*” OR “PD 1 PD L1 blockade*” OR ipilimumab OR strentarga OR yervoy OR nivolumab OR opdivo OR pembrolizumab OR keytruda OR lambrolizumab OR atezolizumab OR tecentriq OR tecntriq OR avelumab OR bavencio OR durvalumab OR imfinzi OR cemiplimab OR libtayo) AND (LIMIT-TO (LANGUAGE , "English")) AND (EXCLUDE (DOCTYPE , "ch") OR EXCLUDE (DOCTYPE , "ed"))

Web of Science (Science Citation Index Expanded, Social Sciences Citation Index, Arts & Humanities Index, and Emerging Sources Citation Index)

= 120 results on 8/18/2021; Results limited to English

Updated search (original search with date limit 2021-present): 34 results on 6/14/2022

TS=((melanoma* OR melanocarcinoma* OR melanomalignoma* OR naevocarcinoma* OR nevocarcinoma*)) AND TS=((“NRAS” OR “NRAS1” OR “NS6” OR “N-ras” OR “neuroblastoma RAS viral oncogene homolog” OR “v-ras neuroblastoma RAS viral oncogene homolog” OR “oncogene N ras”)) AND TS=((“immune checkpoint inhibitor*” OR “checkpoint inhibitor immunotherap*” OR “immune checkpoint block*” OR “immune checkpoint inhabitation*” OR “PD-L1 inhibitor*” OR “PD L1 inhibitor*” OR “anti-PDL 1” OR “programmed death ligand inhibitor*” OR “programmed death-ligand 1 Inhibitor*” OR “CTLA-4 inhibitor*” OR “CTLA 4 inhibitor*” OR “cytotoxic T-lymphocyte-associated protein 4 inhibitor*” OR “cytotoxic T lymphocyte associated protein 4 inhibitor*” OR “PD-1 inhibitor*” OR “PD 1 inhibitor*” OR “programmed cell death protein 1 inhibitor*” OR “PD-1-PD-L1 blockade*” OR “PD 1 PD L1 blockade*” OR ipilimumab OR strentarga OR yervoy OR nivolumab OR opdivo OR pembrolizumab OR keytruda OR lambrolizumab OR atezolizumab OR tecentriq OR tecntriq OR avelumab OR bavencio OR durvalumab OR imfinzi OR cemiplimab OR libtayo))

Cochrane Library

=5 results on 8/18/2021 (All results from CENTRAL Trials)

Updated search (original search with date limit 2021-present): 3 results (CENTRAL Trials) on 6/14/2022

ID Search Hits

#1 MeSH descriptor: [Melanoma] explode all trees 1881

#2 ((melanoma* OR melanocarcinoma* OR melanomalignoma* OR naevocarcinoma* OR nevocarcinoma*)):ti,ab,kw 5732

#3 #1 OR #2 5734

#4 MeSH descriptor: [Neurofibromin 1] explode all trees 1

#5 ((“NRAS” OR “NRAS1” OR “NS6” OR “N-ras” OR “neuroblastoma RAS viral oncogene homolog” OR “v-ras neuroblastoma RAS viral oncogene homolog” OR “oncogene N ras”)):ti,ab,kw 401

#6 #4 OR #5 402

#7 MeSH descriptor: [Immune Checkpoint Inhibitors] explode all trees 23

#8 MeSH descriptor: [Ipilimumab] explode all trees 210

#9 MeSH descriptor: [Nivolumab] explode all trees 501

#10 ((“immune checkpoint inhibitor*” OR “checkpoint inhibitor immunotherap*” OR “immune checkpoint block*” OR “immune checkpoint inhabitation*” OR “PD-L1 inhibitor*” OR “PD L1 inhibitor*” OR “anti-PDL 1” OR “programmed death ligand inhibitor*” OR “programmed death-ligand 1 Inhibitor*” OR “CTLA-4 inhibitor*” OR “CTLA 4 inhibitor*” OR “cytotoxic T-lymphocyte-associated protein 4 inhibitor*” OR “cytotoxic T lymphocyte associated protein 4 inhibitor*” OR “PD-1 inhibitor*” OR “PD 1 inhibitor*” OR “programmed cell death protein 1 inhibitor*” OR “PD 1 PD L1 blockade*” OR ipilimumab OR strentarga OR yervoy OR nivolumab OR opdivo OR pembrolizumab OR keytruda OR lambrolizumab OR atezolizumab OR tecentriq OR tecntriq OR avelumab OR bavencio OR durvalumab OR imfinzi OR cemiplimab OR libtayo)):ti,ab,kw 5843

#11 #7 OR #8 OR #9 OR #10 5846

#12 #3 AND #6 AND #11 5

ClinicalTrials.gov

= 6 results on 8/18/2021

Updated Search: No new results retrieved on 6/14/2022

(NRAS OR “NRAS1” OR “NS6”OR “neuroblastoma RAS viral oncogene homolog”) AND AREA[ConditionSearch] (melanoma OR melanomas OR melanocarcinoma) AND AREA[InterventionSearch] (“immune checkpoint inhibitor” OR “checkpoint inhibitor immunotherapy” OR ipilimumab yervoy OR nivolumab OR opdivo OR pembrolizumab OR keytruda OR lambrolizumab OR atezolizumab OR avelumab OR bavencio OR durvalumab OR imfinzi OR cemiplimab OR libtayo)

2 Supplementary Figures

Supplementary Figure 1. A: Funnel plot with contours at p = 0.10, 0.05, and 0.01. B: Funnel plot with two studies (white circles) imputed by the trim-and-fill method. C: Baujat plot; the squared Pearson residual corresponds to the study's level of contribution to the Q statistic of heterogeneity.

A

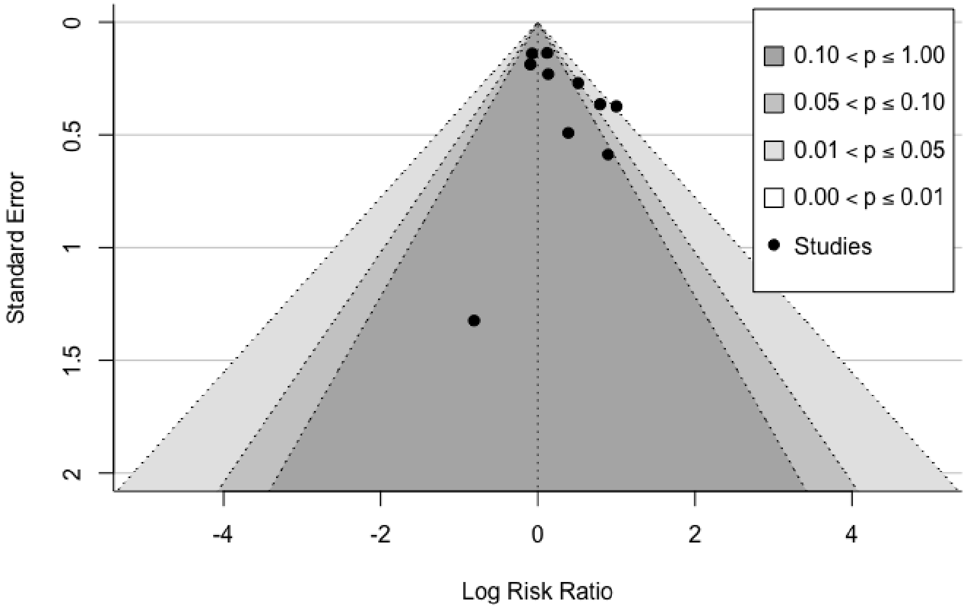

B

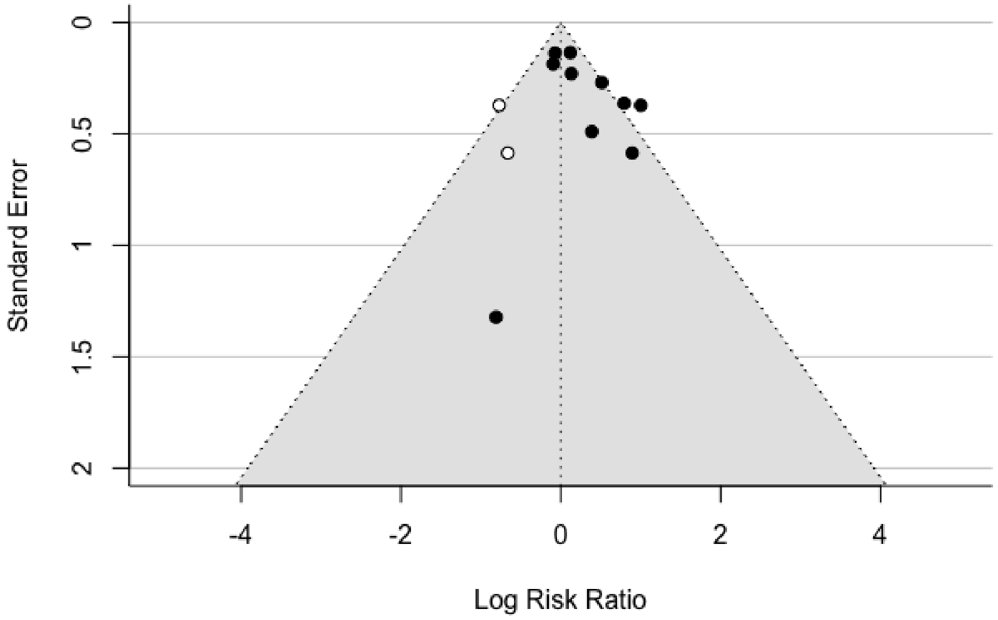

C

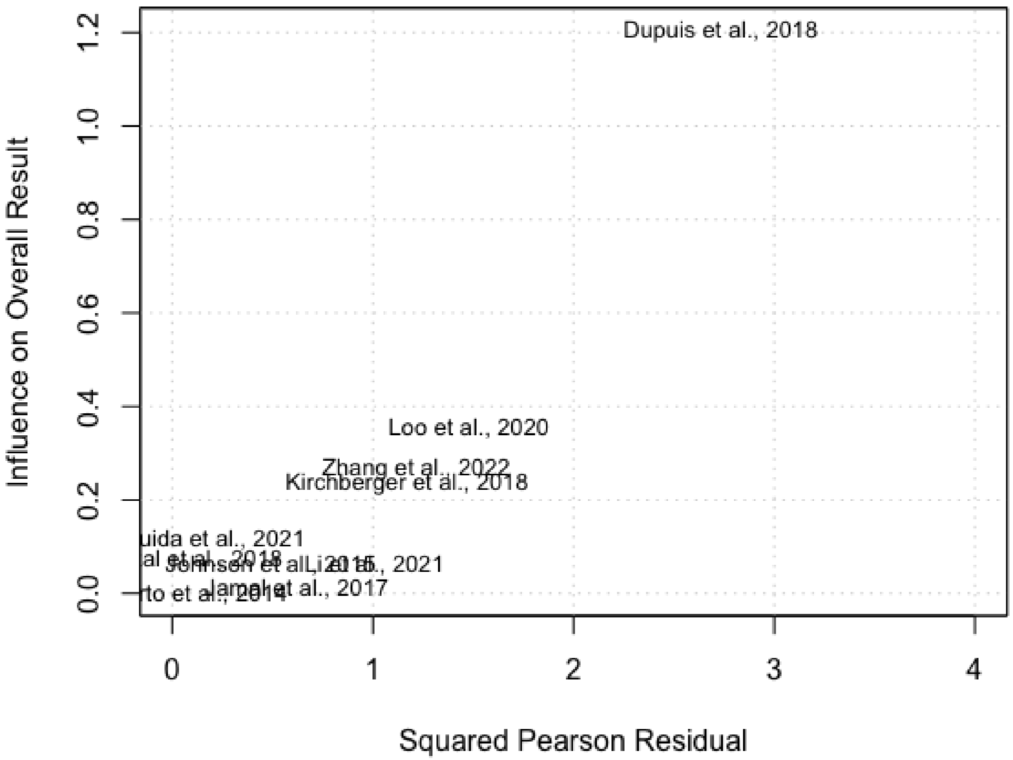
Dupuis et al., 2018
Loo et al., 2020
Zhang et al., 2022
Kirchberger et al., 2018
uida et al., 2021
Li et al., 2021
Jamal et al., 2017
Influence on Overall Result
Squared Pearson Residual
0.0
0.2
0.4
0.6
0.8
1.0
1.2
0
1
2
3
4
